# *Prochlorococcus* as a Possible Source for Transparent Exopolymer Particles (TEP)

**DOI:** 10.3389/fmicb.2017.00709

**Published:** 2017-04-26

**Authors:** Francesca Iuculano, Ignacio P. Mazuecos, Isabel Reche, Susana Agustí

**Affiliations:** ^1^Department of Global Change, Instituto Mediterráneo de Estudios Avanzados, Consejo Superior de Investigaciones Cientificas – Universitat de les Illes BalearsEsporles, Spain; ^2^Departamento de Ecología, Instituto del Agua, Universidad de GranadaGranada, Spain; ^3^Red Sea Research Center, King Abdullah University of Science and TechnologyThuwal, Saudi Arabia

**Keywords:** transparent exopolymer particles, *Prochlorococcus*, Pacific Ocean, Atlantic Ocean, UVR, solar radiation

## Abstract

Transparent exopolymer particles (TEP), usually associated with phytoplankton blooms, promote the formation of marine aggregates. Their exportation to deep waters is considered a key component of the biological carbon pump. Here, we explored the role of solar radiation and picocyanobacteria in the formation of TEP in oligotrophic surface waters of the Atlantic and Pacific Oceans in ten on-deck incubation experiments during the Malaspina 2010 Expedition. TEP concentrations were low on the ocean’s surface although these concentrations were significantly higher on the surface of the Pacific (24.45 ± 2.3 μg XG Eq. L^-1^) than on the surface of the Atlantic Ocean (8.18 ± 4.56 μg XG Eq. L^-1^). Solar radiation induced a significant production of TEP in the on-deck experiments from the surface water of the Pacific Ocean, reaching values up to 187.3 μg XG Eq. L^-1^ compared with the low production observed in the dark controls. By contrast, TEP production in the Atlantic Ocean experiments was lower, and its formation was not related to the light treatments. *Prochlorococcus* sp. from the surface ocean was very sensitive to solar radiation and experienced a high cell decay in the Pacific Ocean experiments. TEP production in the on-deck incubation experiments was closely related to the observed cell decay rates of *Prochlorococcus* sp., suggesting that this picocyanobacteria genus is a potential source of TEP. The evidence to propose such potential role was derived experimentally, using natural communities including the presence of several species and a variety of processes. Laboratory experiments with cultures of a non-axenic strain of *Prochlorococcus marinus* were then used to test TEP production by this genus. TEP concentrations in the culture increased with increasing cell abundance during the exponential phase, reaching the highest TEP concentration at the beginning of the stationary phase. The average TEP concentration of 1474 ± 226 μg XG Eq. L^-1^ (mean ± SE) observed at the stationary phase of *P. marinus* cultures is comparable with the values reported in the literature for diatom cultures, also growing in non-axenic as well as axenic cultures. Our results identify *Prochlorococcus* sp. as a possible relevant source of TEP in the oligotrophic ocean.

## Introduction

Transparent exopolymer particles (TEP) are organic particles composed mainly of acidic polysaccharides (Passow and Alldredge, 1994), and recognized as a key component of the biological carbon pump in the ocean (Engel et al., 2004b; Riebesell et al., 2007; Passow and Carlson, 2012). TEP form marine aggregates that promote the sedimentation of particles and export carbon to deep waters (Passow, 2002a). Their concentration and distribution in the ocean appear to be related to abiotic aggregation from their dissolved or colloidal exopolymeric precursors (Passow, 2000) and biotic processes via exudation from phytoplankton (Kiørboe and Hansen, 1993) and bacterioplankton (Alldredge et al., 1993; Passow, 2002b; Ortega-Retuerta et al., 2010). Formation of TEP has previously been related to phytoplankton abundance and composition (Passow and Alldredge, 1994), with blooms of diatoms (Mari, 1999) and coccolithophorids (Engel et al., 2004a; Van Oostende et al., 2013) the most studied sources. Cyanobacteria blooms have also been reported as sources of TEP (Berman-Frank et al., 2007). However, the role played by picocyanobacteria, the most abundant and ubiquitous primary producers (Partensky et al., 1999) in the open ocean, as a source for TEP remains unclear.

Nutrient limitation and other stresses have been identified as the main drivers of the exudation of dissolved carbohydrates by primary producers (Berman-Frank et al., 2007). Recently, Ortega-Retuerta et al. (2009) demonstrated that ultraviolet B (UVB) radiation stimulated TEP production in the presence of microorganisms, observing a 17% increase in TEP under +UVB treatment in comparison with dark controls. Orellana and Verdugo (2003) reported results in agreement with those of Ortega-Retuerta et al. (2009). However, there are few estimates of TEP distribution in the oligotrophic ocean, although some studies report low concentrations of TEP (Passow, 2000; Ortega-Retuerta et al., 2010). The oligotrophic ocean is characterized by transparent and relatively warm waters and low nutrient concentrations mostly dominated by picophytoplankton (Agawin et al., 2000), including *Prochlorococcus* sp. and *Synechococcus* sp. (Partensky et al., 1999). Oligotrophic waters are exposed to high solar radiation, because of the high transparency of this water (Tedetti and Sempéré, 2006) as well as their location in most subtropical and tropical regions, which attract maximal incident ultraviolet (UV) radiation. Therefore, in the oligotrophic ocean, photo-processes are likely to significantly influence TEP formation (Ortega-Retuerta et al., 2009). Moreover, it is well documented that solar radiation harms picophytoplanktonic cells by inducing cell mortality (Llabrés and Agustí, 2006; Agustí and Llabrés, 2007). In fact, maximum production of TEP has been related to the decline of phytoplankton blooms and to their senescence phases (Passow, 2002b; Engel et al., 2004a). Berman-Frank et al. (2007) identified autocatalytic programmed cell death in *Trichodesmium* sp. as a process inducing TEP production.

In this study, we experimentally tested the hypotheses that solar radiation enhances the production of TEP in the surface of oceanic and oligotrophic waters. We gathered data to test these hypotheses by performing ten on-deck experiments of oligotrophic surface waters from the North Atlantic and inter-equatorial Pacific Oceans during the Malaspina 2010 Expedition. We exposed surface waters to different light treatments, including dark controls, full solar radiation, and ultraviolet radiation removed (-UV) to observe differences in TEP production. We simultaneously analyzed the sensitivity of picocyanobacteria and heterotrophic bacteria populations to solar radiation. The results obtained with natural oligotrophic communities suggested a potential role for *Prochlorococcus* as a source of TEP. We tested this potential role in the laboratory using a non-axenic culture of *Prochlorococcus marinus*, where reduced sources of variability relative to field experiments allow test of the inference pointing at *Prochlorococcus* as a source of TEP. We then compared the TEP concentration values observed in the *P. marinus* culture with those described in the literature for phytoplankton species reported to produce TEP growing in non-axenic and axenic cultures.

## Materials and Methods

### Sampling Site and Study Area

**FIGURE 1:**
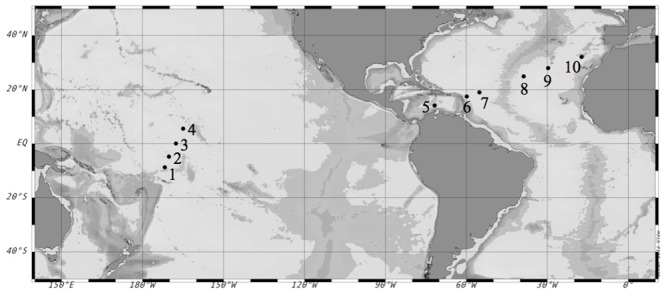
**Location of the selected stations during the Malaspina-2010 Expedition where the experiments were performed.** Circles mark the number of each experiment. Coordinates are reported in **Table 2**.

We conducted 10 on-deck incubation experiments (**Table 1**) on board the R/V Hesperides during the Malaspina 2010 Expedition^1^. The first set of experiments (#1–4) was performed in the Pacific Ocean during the expedition’s leg from Auckland, New Zealand to Honolulu, U.S. during April-May 2011. The second set of experiments (#5–10) was performed in the Atlantic Ocean during the expedition’s leg from Cartagena de Indias, Colombia to Cartagena, Spain during June–July 2011 (**Figure 1**).

**Table 1:** Biogeographical provinces, geographical coordinates, biogeochemical conditions, and *in situ* incident solar radiation doses for each experiment.

Exp.	Longhurst province (lat-long)	T (°C)	Salinity	Chl a (*μ*g L^-1^)	*Proch* (cells mL^-1^)	*Syn* (cells mL^-1^)	*HBA* (cells mL^-1^)	UVB (KJ m^-2^)	UVA+B (KJ m^-2^)	Global radiation (KJ m^-2^)
1	PEQD 7.06° 17I.39°W	28.91	35.48	0.14	3.24E+04	4.05E+03	6.84E+05	39	1276	19696
2	PEQD 3.41°S 169.46°W	28.56	35.36	0.23	8.54E+04	2.90E+03	5.54E+05	44.6	1507	16416
3	PEQD 1.60°S 166.85°W	27.65	35.08	0.31	7.30E+04	2.52E+03	1.45E+06	45.1	1569	26591
4	PEQD 6.99°N I64.37°W	27.74	34.81	0.3	6.58E+04	5.36E+03	1.00E+06	46	1539	24639
5	CARB I4.I6°N 71.67°W	29.22	35.56	0.13	1.54E+04	5.06E+03	2.86E+05	23.9	1348	21252
6	NATR 17.43°N 59.83°W	29.43	35.52	0.14	3.68E+04	2.07E+03	6.72E+05	24.5	1334	21000
7	NATR 19.00°N 55.15°W	28.51	36.61	0.19	2.49E+04	6.82E+03	2.31E+05	23.7	1311	22558
8	NATR 24.84°N 38.71°W	25.65	37.57	0.07	2.04E+04	4.85E+03	6.11E+05	36.7	1663	27105
9	NASE 27.97°N 29.65°W	23.32	37.32	0.05	1.01E+04	2.14E+03	5.32E+05	34.3	1431	22173
10	NASE 32.08°N 17.27°W	21.81	36.7	0.09	4.29E+04	3.39E+03	7.32E+00	40.4	1660	28909

*Exp, number of experiment; T, *in situ* seawater surface temperature (at 3 m depth); Chl *a*, Chlorophyll *a* concentration (at initial incubation time). *Proch, Syn and HBA* represent the cell abundance of *Prochlorococcus* sp. *Synechococcus* sp. and heterotrophic bacteria in seawater, respectively, at the initial incubation time. Incident UV radiation data are expressed as total doses received per day. PEQD, Trades-Pacific Equatorial Divergence; PNEC, Trades-North Pacific Equatorial Countercurrent Province; CARB, Caribbean Province; NATR, Trades-North Atlantic Tropical Gyral Province; NASE, North Atlantic Subtropical Gyral Province (Longhurst, 1998).*

### Experimental Setup

Water samples were collected at 3 m depth in 30 L Niskin bottles. The water was filtered through a 50 μm mesh to remove the largest fraction of grazers. Water samples were dispensed into duplicate 2 L bottles for each treatment: quartz flasks, which allowed the full solar light spectrum (FULL treatment) to pass through, polycarbonate Nalgene bottles, which removed UVB and most UVA, and between 35% of transmittance at 399 nm and 86% of transmittance at 419 nm, allowing visible wavelengths to pass through (-UV treatment), and black bottles, which blocked all solar radiation (DARK treatment). The bottles were incubated in an open-air tank (*V* = 1.9 × 10^3^ L) and maintained at *in situ* temperature by using a continuous surface water running system. The experiments started after sunrise, at about 07:00 h local time (initial time T_0_), and lasted for approximately 24 h (before dawn, final time T_f_). The sampling time at T_0_ and T_f_ were used for Chl *a* and TEP analysis (except for Exp 4 during which extra sampling at T_3_ in the FULL treatment was also performed). Intermediate samplings coupled with the photoperiod were also performed to study picoplankton abundances: a first sampling (T_1_) after 4 h of sunlight exposure, a second sampling (T_2_) after 6 to 8 h, a third sampling at sunset (T_3_) and a fourth sampling (T_4_) 1 h after sunset.

In experiments 4, 5, and 6 an additional treatment to control for potential abiotic TEP production, due to spontaneous assemblage from dissolved acidic polysaccharide precursors was included by incubating seawater filtered by gravity through a 0.2 μm cartridge (to eliminate phytoplankton and bacterial cells) into duplicate 2 L quartz bottles.

### Solar Radiation

Incident solar radiation (W/m^2^) was measured every 2 min by a Radiation Sensor (2770; Aanderaa Instruments) installed in the meteorological station of the vessel (SADO) placed over the deck. The sensor is sensitive in the range of 300–2500 nm, but is covered by a glass (borosilicate) dome, filtering out most of the incident UVR. To measure solar radiation in the ultraviolet bands, we used an incident UVA (315–400 nm) and UVB (280–315 nm) radiometer (UVS-AB-T; Kipp & Zonen, Version T2.07.217) also installed in the vessel’s meteorological station. Doses of solar radiation received during the experiments were then calculated by integrating radiation values for the day’s duration and expressed in kJ/m^2^.

### Transparent Exopolymer Particles

Transparent Exopolymer Particles were stained with alcian blue, a group of basic, polyvalent and water-soluble dyes, derived from phthalocyanine. In a solution of 3% acetic acid (pH = 2.5) alcian blue stained carboxylated and esther-sulfate groups of acidic mucopolysaccharides. TEP were retained on polycarbonate filters (Poretics) with 0.4 μm pore size and of 25 mm in diameter and stained with 1 mL working solution of alcian blue pre-filtered through a 0.2 μm filter to a final concentration of 0.02%. TEP concentrations were determined spectrophotometrically following the colorimetric method proposed by Passow and Alldredge (1995). For each treatment of the experiments, 400 to 1000 mL volumes of the sample (depending on the saturation of the filter) were collected at the initial (T_0_) and final (T_f_) times (in Exp 4 also at T_3_). Duplicates were filtered under low and constant pressure (150 mmHg). Filters were subsequently stained with 1000 μL of a 0.02% working solution of Alcian blue (pre-filtered through a 0.2 μm filter) in 0.06% acetic acid (pH = 2.5), left for few seconds, filtered again and rinsed twice with MilliQ water to eliminate excess dye. The filters were frozen at -80°C and stored until further processing in the laboratory. The filters were then thawed, soaked in 80% sulfuric acid for 2–3 h, and the absorbances were measured by means of a spectrophotometer (PerkinElmer UV-VIS Lambda 35) at a fixed wavelength of 787 nm. Empty stained filters (stained and frozen in parallel with the samples) were used as blanks.

Alcian blue absorption was calibrated using a Xanthan Gum (XG) solution that was homogenized by a tissue grinder and measured by weight. TEP concentrations were, therefore, expressed in μg of XG equivalents per liter and calculated as follows:

(1) $$\begin{array}{c} \mathrm{TEP}\;(\mu g\;\mathrm{XG}\;\mathrm{Eq}.\;L^{-1})\;=\;(a_{\mathrm{sample}}-a_{\mathrm{blank}})V^{-1}\;\times \;F, \end{array}$$

where *a*_sample_ is the absorbance of the sample, *a*_blank_ is the absorbance of the blank, *V* is the filtered volume of the sample and *F* is the calibration factor, which is the inverse of the slope between μg of XG and alcian blue absorption at 787 nm. The detection limit of the method was 1 μg of XG equivalents L^-1^ and the coefficient of variation was ca. 20%.

The TEP production rate per day (α) was calculated as the natural logarithm of the ratio between the concentrations (μg XG Eq. L^-1^) observed at the final and initial experimental times (*t* = 1 day):

(2) $$\begin{array}{c} \;\alpha \;(d^{-1})\;=\;\ln\;(\mathrm{TEP}\;t_{f}/TEP\;t_{0})/t, \end{array}$$

where TEP *t*_f_ and *t*_0_ are the TEP concentrations at the final and initial times, respectively.

We also calculated the normalized (Passow, 2012) or net (Egge et al., 2009; MacGilchrist et al., 2014) TEP production (TEP_PR_) expressed as μg XG Eq. L^-1^ d^-1^ and calculated from the difference between the TEP concentration at the end of the experiment (TEP *t*_f_) minus the initial TEP concentration (TEP *t*_0_) divided by the experimental time:

(3) $$\begin{array}{c} \mathrm{Net}\;{\mathrm{TEP}}_{\mathrm{PR}}\;(\mu g\;\mathrm{XG}\;\mathrm{Eq}.\;L^{-1}\;d^{-1})\;=\;\frac{\left(\mathrm{TEP}\;t_{f}-TEP\;t_{0}\right)}{\left(t_{f}-t_{0}\right)}. \end{array}$$

### Chlorophyll *a*

The concentration of chlorophyll *a* (Chl *a*) was quantified by using the fluorimetric method described by Parsons et al. (1984). Seawater samples (250 mL) were collected at the initial (T_0_) and final (T_f_) time of each experiment and filtered through 25 mm Whatman GF/F filters. Chlorophyll *a* in the filters was extracted in 90% acetone and left 24 h in the dark at 4°C until processing in a Turner Design fluorimeter, calibrated with pure chlorophyll *a*.

### Picoplankton Abundance

Picoplankton populations were quantified by using flow cytometry.

To quantify the abundance of heterotrophic prokaryotes, we fixed 1.2 mL of each sample treatment from all sampling times (T_0_, T_1_, T_2_, T_3_, T_4_, T_f_) with 48 μL of glutaraldehyde (final concentration 1%). The samples were left for 10 min in the dark and then were frozen in liquid nitrogen and stored at -80°C until analysis in a FACScalibur (Becton Dickinson) flow cytometer equipped with a 488 nm argon laser. The samples were defrosted and analyzed following Marie et al. (1997) by staining 400 μL with 4 μL of 10 × SYBR Green I (Molecular probe S-7563) solution (final dilution 1:1000 [vol/vol]) for 10 min and letting the stained samples run through the FACScalibur at low speed (ca. 25 μL min^-1^) and adding 10 μL of a fluorescent 1 μm latex beads solution as an internal standard for cytometric counts. The run terminated when about 10,000 particles had been recorded or after 90 s. The threshold was set in the green channel. Stained bacteria were detected and discriminated from other non-bacterial particles with a light side scatter (SSC) and green (FL1) and red fluorescence (FL3).

To measure the abundance of phototrophic prokaryotes (*Prochlorococcus* and *Synechococcus*), we ran 400 μL of each sample treatment from all sampling times (T_0_, T_1_, T_2_, T_3_, T_4_, T_f_) at medium speed (ca. 55 μL min^-1^) in the flow cytometer. A sample (10 μL) of a calibrated solution of 1-μm diameter yellow-green fluorescent latex beads (Polysciences) was added as an internal standard for the quantification of cell concentrations. Beads concentrations [9.33 × 10^5^mL^-1^ for the Pacific Ocean experiments] [4.63 × 10^5^mL^-1^ for the Atlantic Ocean experiments] in the standard solution were calculated by filtering duplicate aliquots onto black Nucleopore filters and counting under an epifluorescence microscope. All samples were fixed with glutaraldehyde (1% final concentration) and stored at -80°C until their analysis in the laboratory. *Synechococcus* populations were detected by an orange fluorescence dot plot (FL2 = interval between 585 ± 20 nm of wavelength, corresponding to the fluorescence of the phycoerythrin pigment) versus red fluorescence (FL3 ≥ 635 nm, corresponding to the fluorescence of chlorophyll). *Prochlorococcus* had a lower FL3 signal and no FL2 signal. Moreover, the populations were detected in a SSC versus FL3 dot plot (Marie et al., 1997). Decay rates and growth rates (h^-1^) of *Synechococcus, Prochlorococcus*, and heterotrophic bacterial populations were then calculated as the slope of the linear regression between the natural logarithms of the concentrations (cells mL^-1^) and the experimental time (hours) during each sampling interval.

### *Prochlorococcus marinus* Batch Cultures

To corroborate that *Prochlorococcus* could be a source of TEP we set up two non-axenic cultures of a strain of *P. marinus* (RCC 0156). This strain was grown in the laboratory under one-liter batch aseptic conditions with PCR-11 culture medium at 22°C under low light of 30 μmol Photons m^-2^ s^-1^. We did not include any UV light incubation due to the difficulty and limitations of keeping this species growing in the laboratory. The cultures were sampled regularly during 2 months to monitor changes in cell abundance and TEP generation during the exponential and stationary phases. Triplicated volumes of 20 mL for TEP quantification were sampled by syringe, gently filtered under low vacuum pressure and immediately analyzed as previously described (Passow and Alldredge, 1995). *P. marinus* cell abundance (cells mL^-1^) was quantified in replicated 1 ml fresh samples in a BD FACScalibur flow cytometer.

### Statistical Analyses

Statistical analyses of TEP and picoplankton data were carried out using JMP software, Student’s *t*-test was used to test for significant differences between oceans, treatments (DARK, FULL, -UV) and other changes in parameters, and linear regression was used for the analysis of the relationship between TEP production and picoplankton cell decay.

## Results

### Surface Ocean Conditions

Surface temperature and salinity data collected from the stations near the sampling areas indicated that the experiments were conducted in warm waters (**Table 1**), ranging from 29.4°C in the Caribbean Sea to 21.8°C at the most north-eastern station of the Atlantic Ocean (**Figure 1**); the most saline waters were located in the North Atlantic Ocean (**Table 1**).

The chlorophyll *a* concentration was low in all the biogeographic provinces studied, ranging from 0.05 μg L^-1^ in the Northern Atlantic to 0.31 μg L^-1^ in the equatorial Pacific Ocean.

Initial abundances of picoplankton were generally higher in the waters sampled in the Pacific Ocean than in the waters sampled in the Atlantic Ocean. Heterotrophic bacterial concentrations were also higher in the Pacific Ocean (mean 9.23 ± 2.00 × 10^5^ cells mL^-1^) than in the Atlantic Ocean (mean 3.96 ± 1.39 × 10^5^ cells mL^-1^), reaching maximum abundance at the Equatorial station (1.45 ± 0.4.3 × 10^6^ cells mL^-1^). *Prochlorococcus* sp. was more abundant (mean Pacific 6.42 ± 0.38 × 10^4^ cells mL^-1^; mean Atlantic 2.51 ± 0.17 × 10^4^ cells mL^-1^) than *Synechococcus* sp. (mean Pacific 3.71 ± 0.50 × 10^3^ cells mL^-1^; mean Atlantic 4.05 ± 0.33 × 10^3^ cells mL^-1^).

UVR conditions measured during the experiments in the Pacific Ocean were slightly higher at the northern latitudes and were the highest during Exp 4 (**Table 1**). During the Atlantic Ocean experiments, UVR doses were slightly higher at the northern latitudes, in the North Atlantic Gyre, than in the Caribbean experiments but in all the cases lower than during the Pacific Ocean experiments (**Table 1**). UVB daily doses measured during the Pacific Ocean experiments (mean 43.6 ± 3.1 KJ m^-2^) were significantly higher than those measured during the Atlantic Ocean experiments (mean 30.5 ± 2.5 KJ m^-2^; *t* = 13.09, df = 8, *p* < 0.005).

**FIGURE 2:**
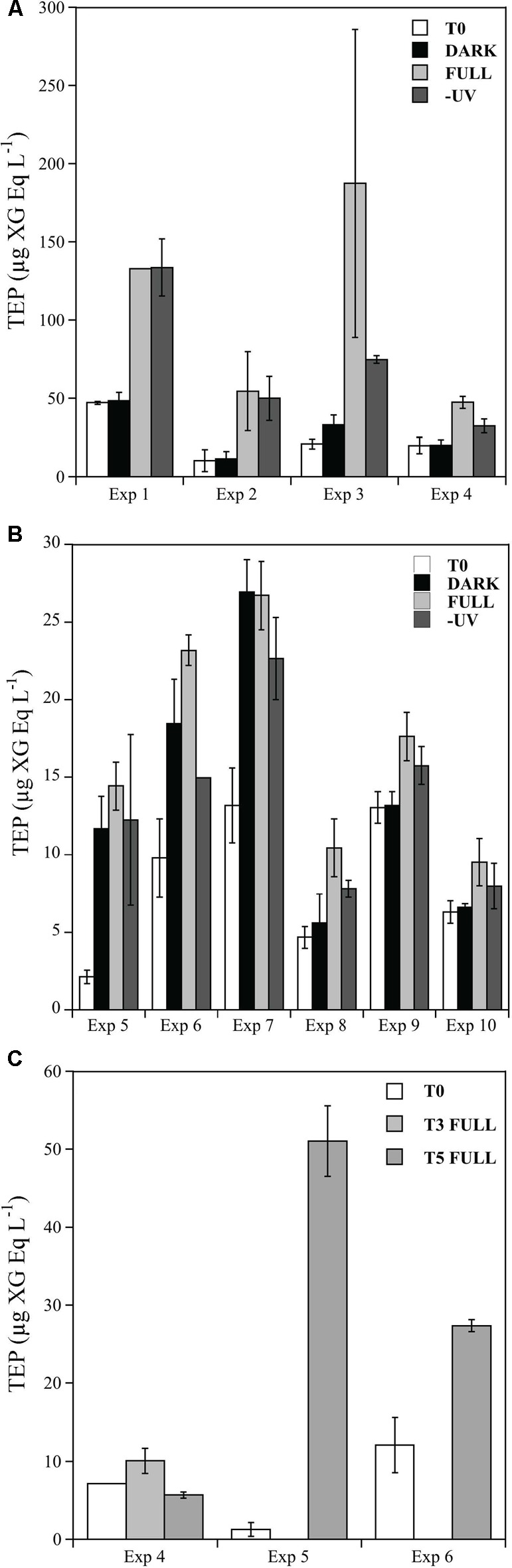
**Changes in TEP concentrations (μg XG Eq. L^-1^) over incubation time for initial time (white bars), DARK (black bars), -UV (gray bars), and FULL (light gray bars) treatments during the four experiments performed in the Pacific Ocean (A)** and the six experiments performed in the Atlantic Ocean **(B)** and in abiotic treatments **(C)**. Error bars represent standard errors of duplicates. Note that **(A,B)** have different *y*-axis scale.

Initial TEP concentrations in the Pacific Ocean were significantly higher (mean 24.45 ± 2.3 μg XG Eq. L^-1^) than in the Atlantic Ocean (mean 8.18 ± 4.56 μg XG Eq. L^-1^; *t*-test: *t* = 16.26, df = 8, *p* < 0.05) (**Figure 2**).

### On-deck Solar Radiation Incubation Experiments

In all the experiments performed in the Pacific Ocean, we detected, after the 24 h of incubation, a significant increase in TEP concentrations under full solar radiation, but not in the DARK and -UV treatments (**Figure 2A**; *t*-test: Full-Initial *t* = 81.05, df = 7, *p* < 0.05). The highest concentration of TEP was consistently observed in the full solar radiation treatments, reaching values up to 187.3 μg XG Eq. L^-1^ in the Exp 3 (**Figure 2A**). The potential that TEP was produced by self-assembly due to abiotic processes was tested in Exp 4. When microorganisms were excluded from the waters, there was no detectable increase in TEP (**Figure 2C**). An additional sampling time at the end of the day in Exp 4 revealed that the production of TEP during the day increased to 60.2 ± 8 XG Eq. L^-1^, although no significantly higher than the value observed after 24 h (**Figure 2A**, 47.4 ± 3.83 XG Eq. L^-1^).

In the experiments performed in the North Atlantic Ocean (Exps 5–10, **Table 1**) we also observed an increase in TEP production after the incubation period in most of the experiments, but, in contrast with the Pacific Ocean experiments, this increase in production was not consistently induced by solar radiation exposure (**Figure 2B**). Maximum concentrations of 26.9 μg XG Eq. L^-1^ were produced during Exp 7 (**Figure 2B**). In the experiments 8, 9, and 10, we observed higher concentrations in the full solar radiation treatments but these increases were not statistically significant (**Figure 2B**). In the two experiments (#5 and 6) in which an extra treatment excluding the microorganisms was performed, we observed contrasting results (**Figure 2C**). In Exp 5 significantly more TEP was produced in the 0.2 μm-filtrated treatment (**Figure 2C**) (ca. 50 μg XG Eq. L^-1^) than in the full treatment including microorganisms (**Figure 2B**) (ca. 10 μg XG Eq. L^-1^). This fact suggests the relevance of abiotic self-assembly, although no difference was observed in the case of the Exp 6 (**Figure 2C**).

**FIGURE 3:**
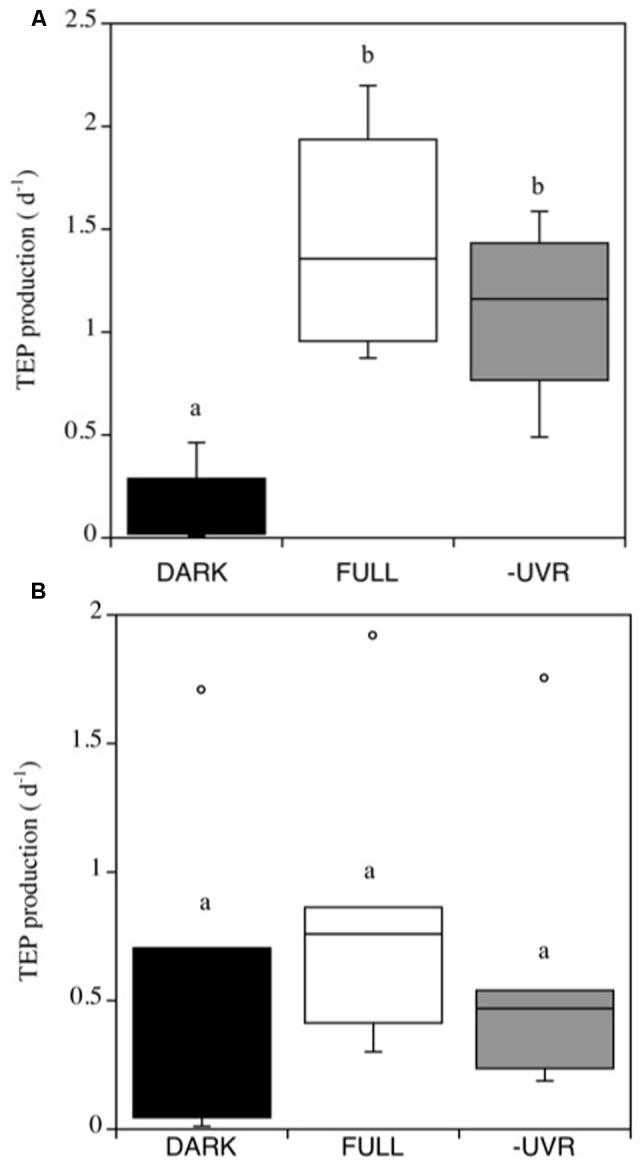
**Transparent exopolymer particles production rates (α, d^-1^) in the Pacific (A)** and Atlantic **(B)** experiments, for the different treatments. FULL: full solar radiation (white box), –UV: UV radiation removed (gray box) and DARK: dark treatment (black box). The boxes show the lower and upper quartiles. The line represents the median value for the experiments, and the caps are the minimum and maximum values. The boxes with the same letter are not significantly different (*p* > 0.05).

Transparent Exopolymer Particles production rates were generally higher in the Pacific Ocean experiments, resulting in a mean net daily TEP production of 44.3 ± 14.2 μg XG Eq. L^-1^, than in the Atlantic Ocean experiments, which averaged 6.6 ± 1.1 μg XG Eq. L^-1^. Furthermore, the TEP production rates (d^-1^) in the Pacific Ocean experiments were significantly higher under the FULL solar radiation treatment (**Figure 3A**). By contrast, TEP production in the Atlantic Ocean did not differ significantly among treatments (**Figure 3B**).

**FIGURE 4:**
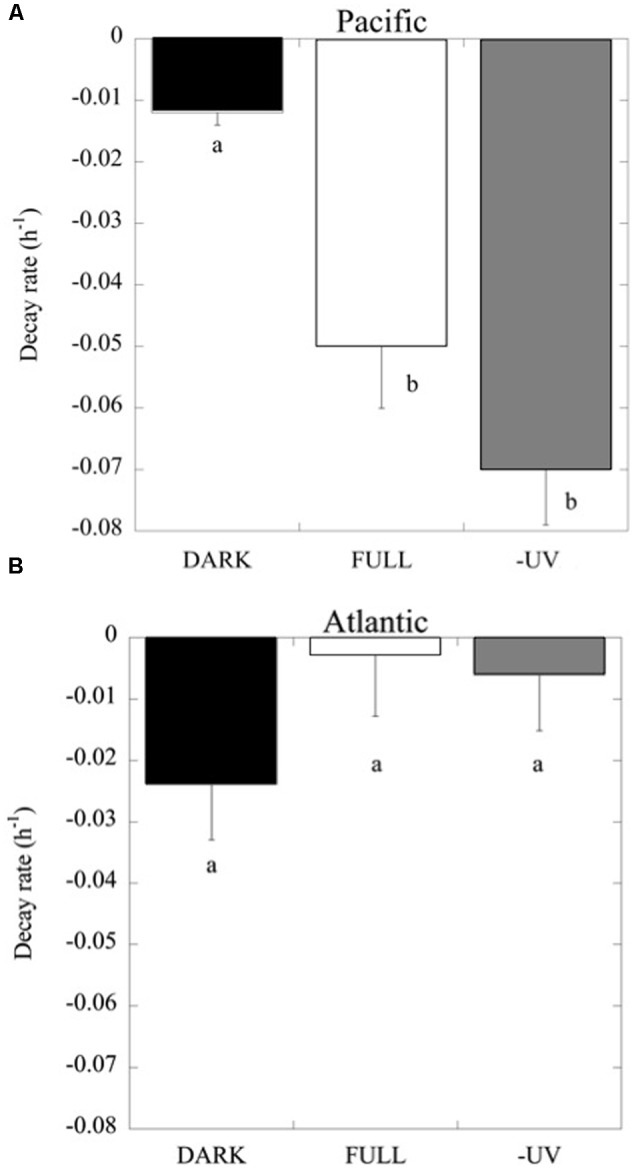
***Prochlorococcus* cell decay rates (h^-1^) at the light treatments FULL, DARK and –UV, as described in **Figure 3**. (A)** Data from experiments with Pacific and **(B)** Atlantic communities. The boxes with the same letter are not significantly different (*p* > 0.05).

**FIGURE 5:**
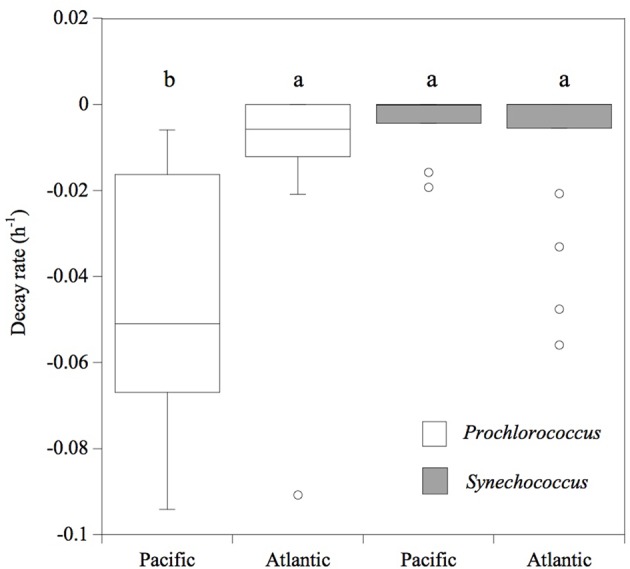
**Cell decay rates (h^-1^) of *Prochlorococcus* (white box) and *Synechococcus* (gray box) cells.** The boxes show the lower and upper quartiles. The line represents the median value for each ocean, the caps are the minimum and maximum values, and the dots are outlier values. The boxes connected with the same letter are not significantly different (*p* > 0.05).

Consistently, in all the experiments performed in the Pacific Ocean, the populations of *Prochlorococcus* sp. were negatively affected by solar radiation (*p* < 0.05), resulting in the decay of the cell population over time (**Figure 4**), without significant differences in the decay rates between FULL (mean -0.06 ± 0.01 h^-1^) and -UV treatments (mean -0.07 ± 0.009 h^-1^), but with significantly higher cell decay rates in comparison with the DARK treatments (**Figure 4**; mean -0.01 ± 0.002 h^-1^; *t*-test: DARK-FULL *t* = 0.058, df = 7, *p* < 0.001; *t*-test: DARK-UV *t* = 0.048, df = 7, *p* < 0.005). The most pronounced decay in cell concentrations during the sampling time was observed during Exp 2. Dark treatments provided a control for other sources of cell mortality than light, including grazing by small ciliates and protists (passing through a net of 50 μm mesh) present in the experimental communities. *Prochlorococcus* decay rates in the dark treatments in the Pacific experiments were low, significantly lower than those in the light treatments. However, in the Atlantic, the differences between dark and light treatments were not significant (*p* > 0.05) and may reflect losses due to other processes. In contrast to *Prochlorococcus*, the *Synechococcus* population exhibited low cell decay rates, which were not significantly different (*p* > 0.05) among treatments (**Figure 5**). Heterotrophic bacterial cell abundance did not decline significantly across the different treatments and experiments in both Pacific and Atlantic experiments. Overall, picoplankton populations from the Atlantic experiments did not exhibit significant decay rates under light exposure treatments except for Exp. 5, which showed the highest decay rates (**Figure 5**). Cell decay of *Prochlorococcus* in the Pacific (mean -0.047 ± 0.009 h^-1^) was significantly higher than in the Atlantic Ocean (mean -0.01 ± 0.005 h^-1^; *t*-test: *t* = 0.036, df = 19, *p* < 0.001) and significantly higher than that of *Synechococcus* in the Pacific Ocean (mean -0.004 ± 0.002 h^-1^; *t*-test: *t* = 0.043, df = 23, *p* < 0.001). However, *Prochlorococcus* and *Synechococcus* decay rates were not significantly different in the Atlantic Ocean (**Figure 5**).

**FIGURE 6:**
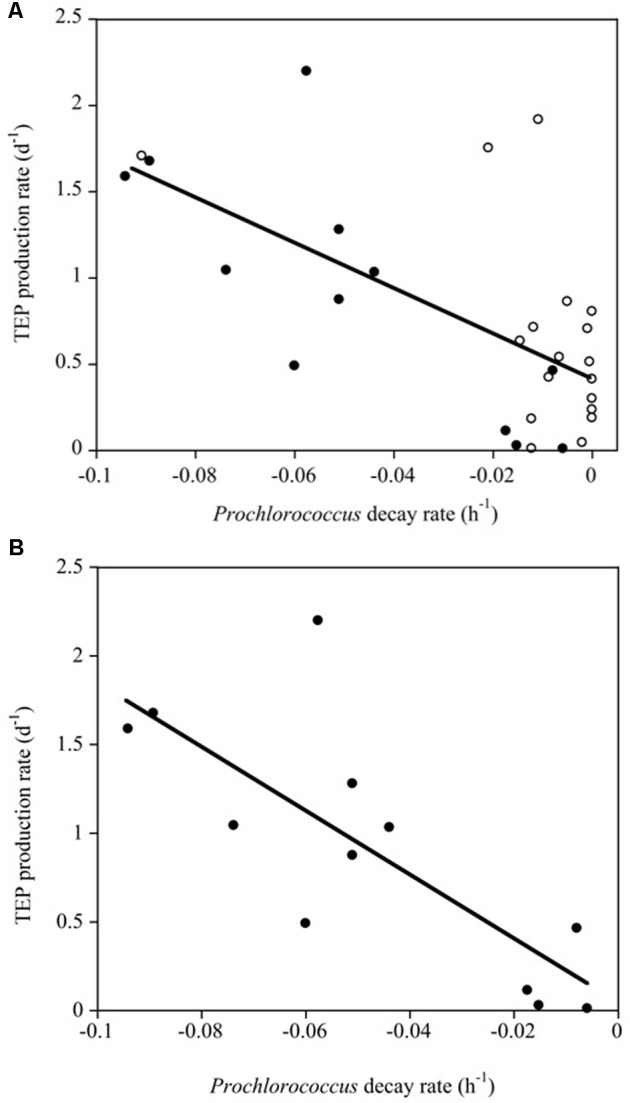
**(A)** Linear regression between TEP production rates (d^-1^) and *Prochlorococcus* decay rates (h^-1^) in the experiments (*R*^2^ = 0.41, *p* < 0.0001). White and black solid dots correspond to data of the Atlantic and Pacific experiments, respectively. **(B)** Linear relationships between TEP production rates (d^-1^) and *Prochlorococcus* decay rates (h^-1^) for the Pacific experiments alone (black dots, *R*^2^ = 0.60, *p* < 0.001).

We did not obtain significant correlations between Chl *a* concentration and bacterioplankton, *Synechococcus* and TEP concentrations or between Chl *a* concentration and TEP production rates (α). However, the TEP production rate (d^-1^) increased significantly with increasing *Prochlorococcus* sp. decay rates (*R*^2^ = 0.41, *p* < 0.0001). This relationship improved when only the Pacific Ocean experiments were considered (α TEP (d^-1^) = 0.047-18.04^∗^*Prochlorococcus* decay (h^-1^), *R*^2^ = 0.60, *p* < 0.001) in which the *Prochlorococcus* cell decay accounted for a larger percentage of the variability (**Figure 6**).

### TEP Concentration and Production in *Prochlorococcus* Cultures

**FIGURE 7:**
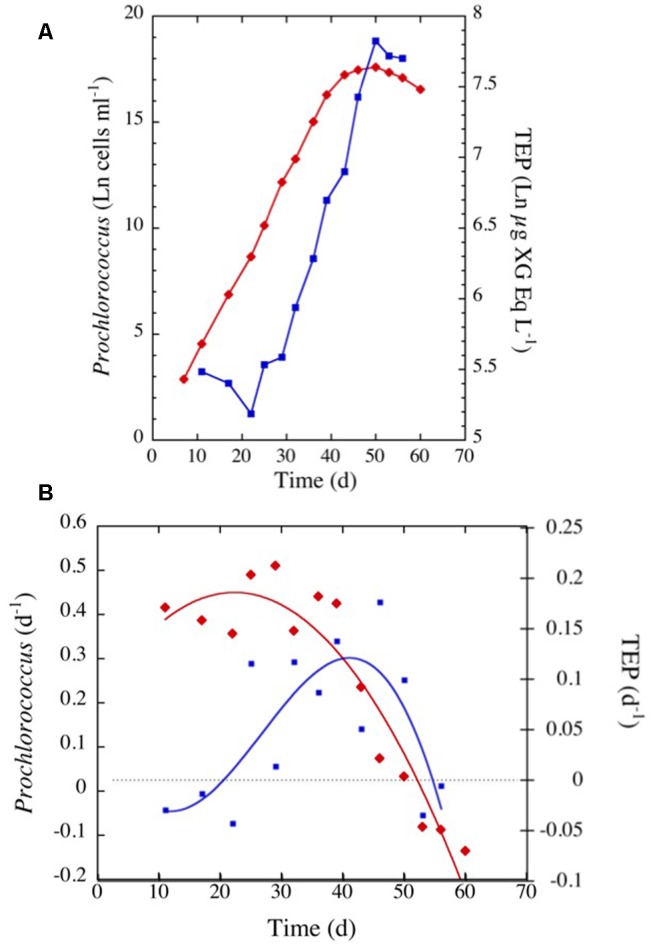
**(A)** Exponential increase with time in TEP concentrations (blue squares and line) and in cell abundance (red diamonds and line) observed in the culture of *Prochlorococcus marinus* under laboratory conditions. **(B)** Changes in *P. marinus* specific growth rate (red diamonds) and TEP production (blue squares) with time. The blue and red lines represent the polynomial fits for TEP (order 5) and growth (order 2) specific rates, respectively.

*Prochlorococcus marinus* in the cultures grew at a moderate rate requiring 40 days to reach the stationary phase (**Figure 7**). TEP concentrations varied highly, increasing with time as *P. marinus* cell abundance increased (**Figure 7A**). The highest concentration of TEP was observed before reaching the stationary phase and remained high during the following days (**Figure 7A** and **Table 2**). TEP production rates in the cultures were low (**Figure 7B**) in comparison with the production rates in the field (**Figure 3**). There was no net production of TEP during the first 3 weeks of the culture although *P. marinus* was growing actively (**Figure 7B**). When growth rates of *P. marinus* declined, TEP production still remained at the highest values (**Figure 7B**). When *P. marinus* experienced negative growth the production of TEP stopped, although the TEP concentration remained high in the culture (**Figure 7**).

**Table 2:** Growth rate (d^-1^, mean ± SE), maximum and mean (±SE) concentration and specific production rates (d^-1^, mean ± SE) of transparent exopolymer particles (TEP) observed in cultures of *Prochlorococcus marinus*.

	*P. marinus* Culture 1	*P. marinus* Culture 2
Maximum TEP (μg XG Eq. L^-1^)	2500	1514
*Growth phase (days 11–40)*		
TEP (μg XG Eq. L^-1^)	295 ± 39	151 ± 19
TEP (d^-1^)	0.048 ± 0.008	0.040 ± 0.05
Growth rate (d^-1^)	0.4 ± 0.02	0.23 ± 0.06
*Stationary phase (days 40–56)*		
TEP (μg XG Eq. L^-1^)	1647 ± 333	1185 ± 208
TEP (d^-1^)	0.055 ± 0.008	0.038 ± 0.003
Growth rate (d^-1^)	-0.10 ± 0.05	-0.07 ± 0.1

## Discussion

Our study showed consistent differences in TEP production on the surfaces of the oligotrophic Atlantic and Pacific Oceans. The results also suggest that the picocyanobacteria *Prochlorococcus* may contribute to the production of TEP in the open ocean, as we observed in the Pacific Ocean experiments under solar radiation stress. This TEP production might affect the exportation of carbon to the deep ocean in oceanic oligotrophic regions.

The values of TEP concentrations from surface waters reported in this study were low, in the range of the lowest reported in the literature for the oligotrophic Mediterranean Sea (Ortega-Retuerta et al., 2010). Passow (2002b) compared values of TEP across different areas and found the values in the North Atlantic Ocean were also lower than those reported in the Pacific Ocean. Wurl et al. (2011) reported higher TEP values for the surface of the North Pacific and tropical North Pacific than those found in our study, although the chlorophyll *a* concentration was also higher in that study and a bloom of *Trichodesmium* sp. was reported. The main differences between the Pacific and the Atlantic Ocean sampling areas in this study were connected to the incident solar radiation doses in the areas. There were significantly higher UVB radiation doses reaching the Pacific Ocean than the Atlantic Ocean. Also, the phytoplankton biomass and the abundance of *Prochlorococcus* sp. were higher in the Pacific Ocean sampling areas than in the Atlantic Ocean sampling areas. The TEP-to-Chl *a* concentration ratio averaged 357.3 ± 126.6 and 78.6 ± 9.3 in the Pacific and Atlantic Oceans, respectively, and it was indeed significantly higher in the Pacific Ocean. Overall, we found that during the experiments conducted in the Pacific Ocean, the production of TEP was associated with high-energy solar radiation exposure (both UV and visible wavelengths), suggesting a potential role of light stress in the production of these exopolymers. By contrast, in the experiments # 5, 6, and 7 of the Atlantic Ocean we did not observe significant differences between the dark and the full treatments, but TEP concentrations were higher than the initial conditions. In these experiments, abiotic self-assembling of exopolymers (Verdugo et al., 2004) or likely aggregation mediated by bacteria carbon processing (Stoderegger and Herndl, 1998; Rochelle-Newall et al., 2010) appear to occur independently of light conditions.

Our study is the first to identify *Prochlorococcus* as a possible source of TEP. Callieri et al. (2011) previously suggested this capacity for freshwater *Synechococcus*, resulting in cell aggregation and colonial formation under high ultraviolet radiation exposure. TEP is also produced by marine *Synechococcus* (Deng et al., 2016). The *P. marinus* cultures also suggest that this species is able to generate TEP directly or indirectly through stimulus of associated bacteria. The TEP concentration increased from the growth phase to the stationary phase, similar to the concentration changes in other phytoplankton species grown in cultures (Passow, 2002a). Most literature data came form non-axenic cultures of phytoplankton rending our *P. marinus* cultures results comparable. Maximum TEP concentrations were observed at the beginning of the stationary phase of *P. marinus* cultures and were in the same order of magnitude of those reported for other phytoplankton species, such as the diatoms *Chaetoceros affinis* and *Nitzschia angularis* (Passow, 2002b). Although other diatomic species are able to produce higher TEP concentrations (Passow, 2002a,b), other phytoplankton species, such as *Emiliania huxleyi* and *Tetraselmis suecica* produced much lower TEP concentrations, on the order of 740 and 880 μg XG Eq. L^-1^, respectively (Passow, 2002a) than did those found in the *P. marinus* cultures. However, as discussed by Passow (2002b) about other phytoplankton cultures, we can not therefore disregard the role of bacteria in TEP production in our batch cultures. Although phytoplankton is considered the major source for the generation of TEP precursors (Passow, 2002a), heterotrophic bacteria were also identified to have an important role in the formation of TEP in the ocean (Stoderegger and Herndl, 1998; Ortega-Retuerta et al., 2010). Recent works by Gärdes et al. (2011) and Van Oostende et al. (2013) have reported that bacteria attached to diatoms or associated with the coccolithophore *E. huxleyi* are ultimately the promoters of sticky exopolymers that aggregate to larger particles. In fact, bacteria appear to uptake the carbohydrates released by phytoplankton cells producing more refractory and stickier TEP as dissolved organic carbon get older (Rochelle-Newall et al., 2010).

Our results relating TEP production with picocyanobacteria cell decay are in agreement with results from Berman-Frank et al. (2007) who documented that TEP production was associated with cell mortality of the cyanobacteria *Trichodesmium*. In their study, natural populations of *Trichodesmium erythraeum* from New Caledonia were exposed to artificial high irradiance to induce cell mortality (autocatalytic programmed cell death, PCD) and TEP formation was observed. The induced oxidative stress activated PCD and positively correlated with increased TEP production (Berman-Frank et al., 2007). In cultures of the same species, the formation of TEP increased during depleted iron conditions, associated with *T. erythraeum* PCD (Berman-Frank et al., 2007). In the North Adriatic Sea, phytoplankton cell lysis was related to extensive exudation of particulate polysaccharides (Baldi et al., 1997). The production of TEP *in situ* has been related to the decline of phytoplankton blooms and their senescence phases (Passow, 2002b; Engel et al., 2004a). Lysis of *Prochlorococcus* cells dying under high solar radiation may also contribute to the release of intracellular substances to the medium, as observed in oligotrophic systems (Agustí and Duarte, 2013) contributing to TEP production directly or indirectly through bacteria processing. Phosphorus nutrition may influence DOC excretion by *Prochlorococcus* (Bertilsson et al., 2005). It has also been reported that TEP production increased as growth rates decreased at the end of diatom or coccolithophorids blooms, with consequences on settling cell aggregates and carbon export (Logan et al., 1995; Engel et al., 2004a). High concentrations of TEP have also been monitored during blooms dominated by *Phaeocystis* spp. (Riebesell et al., 1995), dinoflagellates (Alldredge et al., 1998), coccolithophorids (Engel et al., 2004a), and cyanobacteria in lakes (Grossart and Simon, 1997). However, studies addressing the relationship between TEP production and picophytoplankton in the open ocean have been lacking. Our results describe TEP production rates in the experiments in the Atlantic Ocean to be on the order of those reported by Ortega-Retuerta et al. (2010) in the Mediterranean Sea; however, TEP production rates in the experiments in the Pacific Ocean under intense solar radiation were higher. Solar radiation, including both PAR and UVR bands, induced significant mortality in *Prochlorococcus* sp. in the Pacific Ocean and one experiment in the Atlantic Ocean. *Prochlorococcus* sp. has been described as more sensitive to sunlight damage than *Synechococcus* and picoeukaryotes (Llabrés and Agustí, 2006; Agustí and Llabrés, 2007). Even a short exposure of 30 min to high solar radiation was able to induce cell mortality in *Prochlorococcus* sp. indicating its high sensitivity to UV exposure (Agustí and Llabrés, 2007). UVR also induced the lysis of dead *Prochlorococcus* cells (Llabrés et al., 2010). However, a review of available experiments in the Atlantic Ocean revealed large variability of *Prochlorococcus* sp. cell mortality rates under UVR, varying from non-detectable to high cell mortality rates (Agustí and Llabrés, 2007). The cell decay rates were significantly related to the health condition (i.e., percentage of living cells) of the original population (Agustí and Llabrés, 2007). *Prochlorococcus* sp. sensitivity to UVR would depend, moreover on incident UVR doses, on other factors as nutrients conditions, light history, associated bacteria, and differences in the ability of *Prochlorococcus* populations to use mechanisms to reduce oxidative stress or improve photosystem responses (Morris et al., 2011; Mella-Flores et al., 2012). Therefore, the relative importance of *Synechococcus* sp. in comparison to *Prochlorococcus* sp. in the Atlantic Ocean versus the Pacific Ocean (**Table 1**) could explain, to some extent, the contrasting results observed in the experiments performed in both oceans.

Our study is the first evidence of the association of TEP production with the picocyanobacteria *Prochlorococcus*. In the Pacific Ocean experiments, under high solar radiation, the production of TEP was related with *Prochlorococcus* cell decay. However, this was not a plausible process in the experiments performed in the Atlantic Ocean where both biotic and abiotic processes contributed to the exopolymer particle dynamics. Our results, verified by direct assays in culture experiments, identify the picocyanobacteria *Prochlorococcus* as a possible source of TEP in the oligotrophic ocean. These new insights, despite its limitations, open new research questions arising from our starting hypothesis. Further field and laboratory experiments are required to deepen our understanding on the effect of high light and UVR, as well as on the relevance of *Prochlorococcus* cell death processes in the production of TEP. Moreover, the effect of other drivers already proven to be relevant to TEP dynamics could not be disregarded. Those drivers include abiotic (e.g., nutrient limitation, temperature, pH, aggregation) and biotic processes (e.g., exudation, viral, and grazing pressure) including the tricky on-going debate on the significance of bacteria in TEP production requiring further analysis of bacteria-*Prochlorococcus* interactions. *Prochlorococcus* is the dominant primary producer in the oligotrophic ocean stressing the need to deepen its potential role in the production of TEP.

## Author Contributions

SA, FI, and IR designed the study. FI run the experiments on board. FI and IM analyzed the samples. All authors contribute to writing the manuscript.

## Conflict of Interest Statement

The authors declare that the research was conducted in the absence of any commercial or financial relationships that could be construed as a potential conflict of interest.

## Footnotes

1 http://www.expedicionmalaspina.es
